# Myosins in Osteoclast Formation and Function

**DOI:** 10.3390/biom8040157

**Published:** 2018-11-22

**Authors:** Beth S. Lee

**Affiliations:** Department of Physiology and Cell Biology, The Ohio State University College of Medicine, 304 Hamilton Hall, 1645 Neil Avenue, Columbus, OH 43210, USA; beth.lee@osumc.edu; Tel.: +1-614-688-3585

**Keywords:** osteoclasts, myosin, actin, podosomes, cell fusion, bone resorption

## Abstract

Skeletal quantity and quality are determined by processes of bone modeling and remodeling, which are undertaken by cells that build and resorb bone as they respond to mechanical, hormonal, and other external and internal signals. As the sole bone resorptive cell type, osteoclasts possess a remarkably dynamic actin cytoskeleton that drives their function in this enterprise. Actin rearrangements guide osteoclasts’ capacity for precursor fusion during differentiation, for migration across bone surfaces and sensing of their composition, and for generation of unique actin superstructures required for the resorptive process. In this regard, it is not surprising that myosins, the superfamily of actin-based motor proteins, play key roles in osteoclast physiology. This review briefly summarizes current knowledge of the osteoclast actin cytoskeleton and describes myosins’ roles in osteoclast differentiation, migration, and actin superstructure patterning.

## 1. The Osteoclast Actin Cytoskeleton

**Figure 1:**
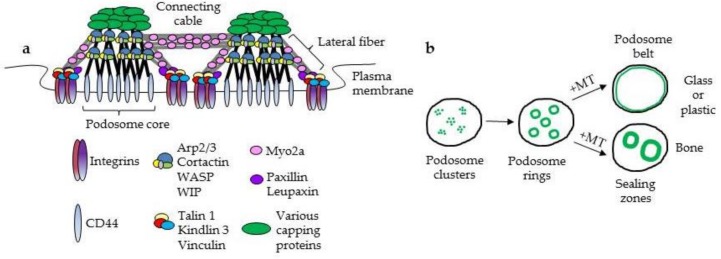
Podosome arrangement in osteoclasts. (**a**) Schematic of selected components of podosomes. Not all proteins indicated are discussed here; their roles in osteoclast physiology are reviewed elsewhere [1]. (**b**) Maturation of podosome assemblies from clusters to rings to belts or sealing zones, depending on the substratum. The latter stages are dependent on microtubules (MT).

Because the actin cytoskeleton plays unique roles in osteoclast formation and activity, its organization and the functions of actin-binding regulatory proteins have been examined in numerous studies [1]. The most marked actin structure that distinguishes osteoclasts is the bone resorptive apparatus, the sealing zone. This structure is an annular gasket-like assembly on the ventral surface of the osteoclast that promotes tight contact with the bone substrate. Enclosed by this ring is the ruffled border, a specialized apical membrane domain that is responsible for secretion of proteases and acid onto the bone surface to promote its degradation. The sealing zone and ruffled border are absolutely required for bone resorption, as even minor disruption of these structures can diminish osteoclast resorptive capacity. The basic building block of the sealing zone is the podosome, an actin-based substrate adhesion assembly that is expressed in a small number of cells, including monocytes, macrophages, smooth muscle, and Src kinase-transformed fibroblasts [2]. When osteoclasts are labeled with fluorescent markers of filamentous or F-actin, podosomes can be visualized as dense dot-like structures (0.5–1.0 µm in diameter) surrounded by more loosely packed actin filaments. The dense labeling represents the core of the podosome, which consists of branched filaments and regulatory proteins oriented perpendicular to the plasma membrane (Figure 1a). The peripheral region, often referred to as the actin cloud, is an assembly of linear actin and regulatory proteins that radiates out from the podosome core to either the plasma membrane (lateral fibers) or neighboring podosomes (connecting cables) [3]. The core filaments attach to the plasma membrane via unknown linkages with the hyaluronate receptor CD44, while the lateral fibers are attached to integrins (dimers composed of α_v_, α_2_, β_1_, β_3_) through associated adaptors and regulatory proteins such as talin, kindlin, and vinculin [4]. Podosomes may also be capped by a variety of proteins that regulate their stability such as formin-like protein 1 (FRL1) and tropomyosin 4 [5,6]. Thus, podosomes may be considered to have two functionally distinct actin domains [4]. The core consists of branched actin filaments whose polymerization is regulated by cortactin, the Arp2/3 complex, Wiskott-Aldrich Syndrome protein (WASp), and WASp-interacting protein (WIP), while actin within the cloud (lateral and connecting cables) appears to be regulated by the formin homology 2 domain-containing protein 1 (FHOD1) [7]. Knockout of WIP results in podosomes lacking the core domain [4], though this can be rescued by matrix-dependent activation of CD44. Conversely, knockout of Src, a cloud regulatory kinase, results in podosomes with reduced or absent clouds [8]. The precise roles of each actin domain in cell adhesion are unclear, as are their roles in podosome formation. However, it has been suggested that clustering of integrins is the first step in podosome assembly, followed by organization of adhesion plaque molecules such as paxillin and the core actin regulatory protein cortactin. This is followed by robust polymerization of F-actin, and accumulation of β_3_ integrin [9]. How CD44 may contribute to assembly is as yet unknown.

The precise configuration of podosome assemblies in osteoclasts depends on the underlying substratum, the maturation level of the osteoclast, and whether or not the cell is actively resorbing bone. Podosomes are able to sense surface stiffness, topography, and to some extent, composition based on the presence of matrix proteins that can engage integrins [10,11,12]. The variety of substructures within and connecting individual podosomes allows for the sensing and generation of mechanical forces at the plasma membrane. This was noted early in studies of osteoclasts, since cells on a highly rigid, smooth substrate like glass or plastic were shown to form different podosome superstructures than cells on similarly rigid but topographically more complex bone [13,14,15]. Osteoclasts are large multinucleated cells that arise from fusion of mononuclear precursors following exposure to soluble factors M-CSF (macrophage colony stimulating factor) and RANKL (receptor activator of NFκB ligand) [16]. As multinucleated osteoclasts begin to form from their precursors, podosomes are first patterned into internal clusters. As maturation proceeds, these clusters become small, short-lived internal rings. When the osteoclasts are on glass or plastic, these rings expand into an interconnected belt of podosomes at the periphery of the cell (Figure 1b). In contrast, when the cells are on bone, the podosome assemblies expand into the thickened, more heavily interconnected sealing zones [3,17,18]. The expansion of internal podosome rings into either a podosome belt or sealing zones is dependent on microtubules, since loss of microtubule integrity causes a collapse of podosome patterning [19,20]. While podosome belts are stable, sealing zones are dynamic structures that dissipate and reform as osteoclasts progress through subsequent cycles of migration and resorption.

Not surprisingly, the changes in this highly dynamic cytoskeleton require activity of the Rho family of small GTPases [21]. These GTPases are critical regulators of the actin cytoskeleton that include the well-characterized subfamily prototypes RhoA, Rac1, and Cdc42, which switch between active, GTP-bound forms and inactive, GDP-bound forms. In their active states, Rho GTPases signal downstream alterations in actin organization. Activation is induced by guanine nucleotide exchange factors (GEFs) that promote release of GDP and subsequent binding of GTP, while GTPase-activating proteins (GAPs) promote hydrolysis of the bound GTP into GDP. The human genome contains dozens of GEFs and GAPs that work together in spatially and kinetically distinct Rho activity domains to permit fine-tuning of cellular actin substructures [22,23]. The entire resorption cycle of osteoclasts requires fine spatiotemporal activation of a variety of Rho family members, and some of the GEFs required in this process have been identified [21,24,25,26]. As will be described below, one of the myosins critical to osteoclast function is also a GAP with specific inhibitory activity toward RhoA, which is required to maintain appropriate podosome and sealing zone organization [27].

## 2. Myosin Classification and Structure

Myosins compose a large superfamily of proteins encoded by different genes. Although they can differ significantly in size, all myosins have a similar structure due to their roles as actin-based motor proteins. At the N-terminus, myosin heavy chains contain a catalytic, actin-binding motor (head) domain, a flexible lever-arm (neck) domain that contains up to six IQ calmodulin-binding motifs, and a C-terminal tail domain that accounts for the bulk of both the structural and functional variability among the myosin forms. Myosins additionally are composed of light chains or calmodulins that bind the neck region of the heavy chain and are involved in stabilization and activation of the myosin complex. Thirty-five classes of myosins are present in the eukaryotic phylogenetic domain [28], and each of these is represented by a Roman numeral (e.g., classes II, VI, XVIII). Further, individual classes may contain multiple genes with distinct functions that are represented by a number and a letter (e.g., Myo1c or myosin IC). Among the 12 classes expressed by humans (I, II, III, V, VI, VII, IX, X, XV, XVI, XVIII, XIX) are 38 myosin genes.

Within the highly conserved head domains of all myosins are actin and ATP binding sites. Hydrolysis of ATP by an actin-activated ATPase within this domain provides the energy for movement of myosins along filamentous actin. However, myosins can vary in the kinetics of this process, the length of time that their head domains are attached to actin filaments (the duty ratio), and the force and movement that can be generated by each ATP hydrolysis cycle. This leads to variation in speed of movement and processivity, or the ability of myosins to remain attached to actin filaments across multiple steps of the catalytic cycle. The details of the physical and mechanical properties of myosin classes are described elsewhere [29,30].

Class II myosins were the first to be discovered because of their predominance in skeletal muscle and their role in muscle contraction [31]. These proteins are able to dimerize and, uniquely among myosins, form bipolar filaments through tail-to-tail oligomerization. Because of the spatial relationship between myosin filaments and F-actin, class II myosins can power actin filament sliding and cellular contraction. Due to their early discovery, class II myosins of both muscle and nonmuscle forms were termed conventional, with all other myosin classes termed unconventional. However, it is now clear that the unconventional myosins far outnumber the conventional forms and are involved in a wide variety of cellular processes. These include carrying intracellular cargo, organization of actin dynamics, actin–microtubule interactions, and regulation of gene transcription, among others [32].

## 3. Class II Myosins

Myo2a (also called myosin IIA or NMIIA) is a nonmuscle myosin with homology to class II muscle myosin. It contains two heavy chains whose tails wrap around each other in a coiled-coil configuration, and four myosin light chains. Phosphorylation of regulatory light chains by nonmuscle myosin light chain kinase (MLCK) activates class II myosin activity while dephosphorylation by myosin phosphatase inhibits it [33]. Like muscle myosin, Myo2a can form fibers and align along actin filaments to generate contractile force, and as such, is a key component of cellular stress fibers [34,35]. The Myo2a heavy chain, encoded by the *Myh9* gene, is a member of the nonmuscle myosin II subfamily whose other members include the Myo2b heavy chain (encoded by *Myh10*; also called myosin IIB or NMIIB) and the Myo2c heavy chain (encoded by *Myh14*; also called myosin IIC or NMIIC). These three nonmuscle class II paralogs have different kinetics, intracellular distributions, and functions [30]. An important role for class II myosins, or indeed any myosin, in osteoclast function was first demonstrated in 1990 when Sato and Grasser generated polyclonal antibodies to myosin II purified from rat liver and microinjected these antibodies into rat osteoclasts [36]. The myosin II used as antigen in these experiments was undoubtedly a mix of nonmuscle Myo2a, Myo2b, and Myo2c since the purification scheme did not select for specific paralogs. Indeed, the existence of multiple nonmuscle class II myosins was not yet well established since these experiments were roughly concurrent with experiments identifying the existence of more than one form [37,38]. Of the three antibodies tested, two significantly reduced myosin ATPase activity and inhibited osteoclast spreading, and all three were able to inhibit the ability of osteoclasts to resorb bone. Further studies on osteoclast myosins did not proceed for more than a decade, when antibodies specific for Myo2a and Myo2b were used to localize the distribution of these myosins in mouse osteoclasts [39]. This study revealed that Myo2a and Myo2b had very distinct patterns of labeling, with Myo2a being associated with the dynamic actin structures of osteoclasts (i.e., podosomes and sealing zone) while Myo2b was excluded from these structures and was generally diffuse in its distribution except for a presence at the cell periphery. In addition, treatment of cells with 2,3-butanedione monoxime (BDM), a somewhat nonselective myosin II inhibitor, rapidly induced detachment of these cells from their substrate, consistent with the involvement of class II myosins in osteoclast adhesion.

This study also provided the first indication that Myo2a may play a role in regulation of podosome patterning. Treatment of osteoclasts with an arginyl-glycyl-aspartic acid (RGD)-containing fragment of osteopontin caused redistribution of podosomes from the cell periphery to clusters within the cell interior [39]. Concurrently, Myo2a became prominent within actin fibers that connect the podosomes, suggesting that it may generate a contractile force to organize these clusters upon sensing of an appropriate substrate. This phenomenon was recently explored in great detail, when modern imaging techniques were used to examine structural components that regulate spatiotemporal organization of podosome clusters in dendritic cells [40]. This study found podosome clusters to be coordinated units that respond to microtopographical cues. As in the earlier study, Myo2a was found to be present in connecting cables that link podosome cores, and reduction of Myo2a activity via various inhibitors (e.g., the class II myosin inhibitor blebbistatin, the myosin light chain kinase inhibitor ML7) caused loss of coordinated movement of podosome components. Therefore, Myo2a appears to play a contractile function in regulating movement of clustered podosomes. Consistent with these findings, another study of podosome patterning in Src-transformed fibroblasts indicated that myosin II activity is required for fission of podosome rings (or rosettes) into daughter rings [41]. Additionally, specific knockdown of Myo2a in osteoclasts with small interfering RNAs (siRNAs) showed that the loss of Myo2a resulted in increased cell spreading with diminished motility and expansion of the sealing zone [42]. These results are consistent with Myo2a generating contractile forces between podosomes throughout the cell. This contractility appears to require the integrin-binding protein paxillin, as direct binding of paxillin to Myo2a was described, and paxillin-deficient osteoclasts exhibit a spreading phenotype similar to cells lacking Myo2a [43]. Further, studies of macrophage podosomes demonstrated that the capping protein supervillin directly interacts with Myo2a to regulate podosome lifespan [44]. A similar role for supervillin in osteoclasts has not yet been confirmed.

Podosomes can be generated on both rigid and soft substrates; however, the configuration of podosomes relies on a combination of factors, including substrate rigidity and topography [45]. Osteoclasts are able to form their resorptive apparatus on bone and other rigid substrates such as calcite, but not on similarly rigid glass or plastic [46]. The precise mechanisms behind the osteoclast’s ability to form sealing zones are still being characterized, but Myo2a contractility appears to play key roles in sensing substrate microtopography and rigidity. Rigidity is an innate property of the substrate, and podosomes are able to detect this property through force generated by cyclical changes in stiffness that are based on polymerization and elongation of their F-actin core [47]. Growth of the core induces both outward protrusions of the plasma membrane and counter force on the lateral actin fibers and associated proteins [48,49]. Generation of tension on the lateral actin fibers via Myo2a-mediated contractility appears to be an important component of this process, since inhibition of Myo2a activity with blebbistatin or siRNAs reduces podosome oscillations and protrusion forces [47,48,49]. In addition, topographically rough surfaces such as native bone promote formation of stable sealing zones, while smooth surfaces tend to promote small, unstable actin rings [11,46,50]. Because of Myo2a’s presence in the connecting cables that join podosomes, it seems likely that tension created by its contractile function would contribute to the ability of linked podosomes to sense substrate discontinuities [12].

In addition to roles for Myo2a in mechanosensing and podosome patterning, targeted knockdown of this myosin in osteoclasts produced an additional, unexpected finding—a role in regulating osteoclast fusion. Specifically, it was determined that downregulation of Myo2a is required, and indeed is sufficient, for stimulating osteoclast precursor fusion [42]. During osteoclastogenesis, levels of Myo2a protein were shown to transiently decrease during the period in which precursor fusion is initiated (approximately 3–4 days following addition of RANKL). This decrease was caused by Myo2a degradation due to a concurrent increase in activity of the protease cathepsin B. Further, siRNA-mediated knockdown of Myo2a increased osteoclast fusion while overexpression of Myo2a inhibited it [42]. These results suggest that high levels of Myo2a inhibit osteoclast fusion, and that its loss triggers the fusion process. Other studies have confirmed these findings [51,52] and have likewise shown that reducing myosin II activity by inhibition of myosin light chain kinase similarly triggers osteoclast fusion [53]. Precisely how Myo2a achieves this inhibition is unclear, but it may be due to production of intracellular tension at defined locations within the cells. 

The process of osteoclast fusion can be initiated by various forms of cell contact [54,55,56]. Many of these fusion events occur when osteoclast precursors tightly interact at a broad contact surface [55]. It has been reported that a unique actin superstructure, the zipper-like structure (ZLS), is transiently formed at these contact sites [53,57]. According to these reports, the ZLS is a thick assembly that is present in partner cells during contact and has structural similarities to the adhesion zipper of epithelial cells [58]. This structure appears to arise from podosome belts at the points of contact and to dissipate as cell–cell fusion occurs [57]. However, the precise composition of the proposed ZLS is unclear and may result from transient podosome-based structures at contact sites. Nonetheless, the authors reported that retrograde flow of actin (i.e., from the cell periphery inward) at this region may push the cells’ plasma membranes outward toward their partners to maintain tight interactions and promote cell fusion [53]. Associated with this region are Myo2a-containing actin filaments that run parallel to the broad cell contact. It is hypothesized that myosin-based contractility in these parallel filaments may counterbalance the membrane protrusive forces initiated by the retrograde actin flow [53]. If so, loss of Myo2a activity could tilt the balance toward cell fusion. However, more experimentation is required to clearly define how Myo2a and its loss might regulate the fusion process.

Other studies have suggested that the phosphorylation state of Myo2a contributes to its regulation of cell fusion. Intercellular interactions between two Ig superfamily members, CD47 and signal regulatory protein alpha (SIRPα; also called SHPS-1), have been shown to stimulate osteoclast precursor fusion. In the absence of CD47/SIRPα binding, osteoclast fusion is strongly inhibited and bone quality is altered [59,60]. Interestingly, CD47/SIRPα binding triggers a dephosphorylation cascade whose targets include Myo2a phosphotyrosines [61]. In macrophages, the phosphorylation status of Myo2a determines its distribution at the plasma membrane, and it is possible that this mechanism plays a key role in the osteoclast fusion process by similarly regulating Myo2a recruitment to the cell membrane. In another set of experiments, the lectin galectin-3 (Gal-3) was shown to play a positive role in osteoclast fusion [52]. A lack of Gal-3 function caused by knockdown or use of an antagonist inhibited the fusion process. Interestingly, Gal-3 was also shown to colocalize at fusion sites with Myo2a and to associate with this myosin in co-immunoprecipitation experiments. Both endogenous, secreted Gal-3 and exogenous Gal-3 associated with Myo2a. It is not yet clear how the extracellular lectin is internalized to associate with Myo2a inside the plasma membrane, nor how Gal-3 might modulate Myo2a activity, so more studies are necessary to understand the ramifications of this association.

Finally, co-opting of Myo2a-dependent fusion pathways by pathogens can result in increased osteoclastogenesis and bone destruction. *Porphyromonas gingivalis* is a bacterial oral pathogen that plays a critical role in promoting alveolar bone resorption in periodontal disease [62]. This microorganism produces the sphingolipid phosphoglycerol dihydroceramide (PGDHC), which is found in substantial amounts in inflamed periodontal tissues. It was reported that PGDHC is cell membrane permeant and can enter osteoclast precursors where its presence stimulates osteoclast fusion. Surprisingly, PGDHC was found to associate with Myo2a in osteoclasts (via pull-down assay followed by mass spectroscopy) and to suppress its inhibitory effects on cell fusion [51]. Specifically, PGDHC was able to reverse the inhibitory effects of Myo2a overexpression on fusion. PGDHC also upregulated expression of dendritic cell-specific transmembrane protein (DC-STAMP) via a Rac1-mediated pathway. Because DC-STAMP is an important positive mediator of osteoclast fusion [63,64], the authors hypothesized that PGDHC exerts its effects through a Myo2a/Rac1/DC-STAMP pathway.

These studies demonstrate that Myo2a plays varied and critical roles in osteoclast formation and function, including regulation of precursor fusion, cell motility, and podosome patterning. Although Myo2b is expressed in osteoclasts, its roles in osteoclast biology have gone unstudied. However, because it appears to be excluded from the regions of podosome organization, it may play a more housekeeping role than one involved in the specialized resorptive activity of osteoclasts. Similarly, Myo2c’s potential functions in osteoclast biology are not yet characterized. However, a recent study performed cytosolic proteomic profiling of monocytes from male osteoporosis patients, and revealed that *MYH14*, the Myo2c heavy chain gene, was one of sixteen genes to show significantly upregulated expression in patients with low bone mineral density [65]. Analyses of other datasets revealed that *MYH14* showed association with low bone mineral density at the proteomic, transcriptomic, and genomic levels [65]. These results suggest that Myo2c may play a crucial role in osteoclastic formation or activity, although no follow-up studies of its physiological role in osteoclasts has yet been performed.

## 4. Myosin X

Class X myosin consists of a single member, Myo10. Important features of this protein include its motor head domain, a lever-arm neck domain containing three IQ domains and a single alpha helix (SAH), a coiled-coil domain, and a long tail with multiple binding capabilities. This tail contains three PH (pleckstrin homology) domains that bind the membrane phospholipid PI(3,4,5)P_3_, and a MyTH4-FERM domain (myosin tail homology domain 4; band 4.1, exrin, radixin, moesin) that binds microtubules. The IQ domains bind calmodulin or calmodulin-like protein. Myo10 dimerizes via its coiled-coil regions in an antiparallel manner that may allow it to walk in a straddle-like fashion on adjacent actin filaments in actin bundles, in addition to walking on single filaments [66,67]. Myo10 was first noted for its prominence at the tips of filopodia, which are long, thin protrusions of the plasma membrane filled with actin filament bundles [68]. Myo10 can move rapidly along the length of filopodia, and indeed, its structure favors movement along bundled actin filaments rather than single chains [69]. Further, Myo10 itself can potently stimulate formation of filopodia as determined by knockdown and overexpression studies [70]. In addition to its role in filopodia formation and transport, Myo10’s ability to bind microtubules places it at the nexus of two distinct cytoskeletal systems. Through this linkage of the actin and microtubule systems, Myo10 is involved in positioning of meiotic and mitotic spindles and regulation of spindle length [71,72,73,74]. Thus, by virtue of Myo10’s role in filopodia formation, it can play broad roles in cell adhesion and signaling, while its regulation of spindle formation demonstrates clear involvement in cell division [75].

Myo10 plays distinct roles in both osteoclast formation and activity. Experiments on differentiated osteoclasts showed that Myo10 is not present in mature podosome belts or sealing zones, but is involved in their patterning [76]. As described above, maturation of these structures into their final expanded forms is a microtubule-dependent process, and Myo10’s ability to act as a linker between the podosomes and microtubules is crucial to the process. When mature osteoclasts containing podosome belts are subjected to cold for several hours, their microtubule structures depolymerize and their podosome belts collapse. The podosome belts are reformed within 24 h by transitioning through the podosome cluster and ring stages before final maturation of the belt, recapitulating the process that occurs during osteoclast maturation. However, suppression of Myo10 expression in osteoclasts by siRNA inhibits the ability of podosome belts to reform after cold treatment. Instead, podosomes are present only in clusters or small rings, indicating that Myo10 is required for the final microtubule-dependent step in podosome belt formation [76]. Similar results were obtained when only the Myo10 MyTH4-FERM domain was overexpressed in cells, since this domain bound microtubules but could not associate with podosomes. Further, in cells with immature podosome rings, immunofluorescence labeling showed Myo10 to be sandwiched between the ring and a layer of microtubules. This, along with coimmunoprecipitation studies, demonstrated how Myo10 physically acts as a linker between the two cytoskeletal systems. Similar results were seen when sealing zones were examined; both siRNA-mediated knockdown of Myo10 and overexpression of the MyTH4-FERM domain led to decreased sealing zone spreading and bone resorptive capacity [76]. These studies therefore demonstrate how this myosin contributes to patterning of the osteoclasts’ most important actin structures.

More recent work examined the role of Myo10 in osteoclast differentiation. In a study of actin structures involved in cell fusion, fusopods were shown to extend between osteoclast precursors prior to fusion [56]. Fusopods were defined as broad structures (width >2 µm) that stained for Myo10 and were enriched in filopodia. These structures, formed from filopodia precursor structures, were abundant in fusion events and had an average length of 2.5 ± 0.2 µm. Because Myo10 was present at the tips of these fusopods as it is in filopodia, it seems likely that this myosin may be critical for fusopod formation and subsequently for cell fusion. This possibility was explored more recently in a separate set of studies. First, in examining Myo10 expression, it was observed that stimulation of differentiation by treatment of precursors with RANKL triggered a large increase in Myo10 expression, and this increase could be amplified further by the additional presence of bone morphogenetic protein 2 (BMP2), another stimulator of osteoclast differentiation [77,78]. Notably, osteoclast precursors treated with lentivirus that expressed shRNAs targeting Myo10 were inhibited in their ability to fuse into multinucleated cells [78]. In addition, expression of several genes critical to osteoclast differentiation was downregulated in these cells, including *Nfatc1*, a master regulator of the osteoclast differentiation program, and *Dc-stamp*, which plays a key role in precursor fusion. The cells were also defective in Smad 1/5/8 activation, which is necessary for responsiveness to BMP signaling and is thought to be involved in a signaling amplification loop involving Myo10 [79]. Further, cells deficient in Myo10 showed a marked loss in the ability to form tunneling nanotubes (TNTs) [78]. TNTs are long, thin, actin-based connections between two cells that do not touch the substratum and are 50–200 nm in diameter and up to 200 µm in length. They were first described in the PC12 cell line [80], but have since been described in many cell types, including monocytes, macrophages, and differentiating osteoclasts [81]. Tunneling nanotubes enable transport of materials between cells, including signaling molecules, small organelles, and even viral pathogens. These structures were found to connect osteoclast precursors early in the differentiation process and to be essential for osteoclastogenesis [82]. In neuronal cells, they previously had been demonstrated to arise from Myo10-driven dorsal filopodia, and their formation required Myo10 [83]. Similarly, the presence of TNTs in differentiating osteoclasts was significantly diminished with the loss of Myo10 [78]. The relationship between TNTs and fusopods identified in the earlier study is not known. It is possible that TNTs may derive from the shorter, broader fusopods, or they may form independently through different mechanisms. Nonetheless, these data reveal that Myo10 plays key roles in multiple aspects of osteoclastogenesis, as well as podosome and sealing zone patterning in mature osteoclasts.

## 5. Myosin IXB

Because osteoclasts possess unique and dynamic actin cytoskeletons that are regulated by RhoA, it was of interest to determine whether class IX myosins are key to osteoclast cytoskeletal organization. The two members of this class, Myo9a (the orthologue of rat Myr7) and Myo9b (the orthologue of rat Myr5), are unusual in containing a RhoGAP domain in their tail regions. This GAP activity is specific toward Rho (particularly RhoA) with little to no activity toward the related Rac and Cdc42 GTPases [84,85]. Myo9a is expressed primarily in brain, testis, and spleen, while Myo9b is most highly expressed in leukocytes of myelocytic origin [86,87]. Accordingly, osteoclasts express Myo9b but not Myo9a [27]. In addition to the RhoGAP domain, class IX myosins contain four IQ domains within the neck region and an insertion within the motorized head domain that acts as a tether to actin filaments to increase its processivity [87,88]. This insertion also binds calmodulin, and calcium binding here regulates Myo9 ATPase activity and movement along actin filaments, thus suggesting a link between intracellular calcium signaling and Myo9 processivity [89].

RhoA drives podosomes into clusters and rings (rather than the peripheral belt) when osteoclasts are on glass and stimulates sealing zone formation when osteoclasts are on bone [90]. Conversely, a podosome belt is formed on either substrate when RhoA activity is minimal. Consistent with its function as an inhibitor of RhoA, Myo9b in osteoclasts was found to be enriched in locations of low RhoA activity (podosome belts) and absent from regions of high RhoA activity (sealing zones) [27]. siRNA-mediated knockdown of Myo9b in mature osteoclasts resulted in increased overall RhoA activity, decreased migration, and altered podosome patterning. Specifically, cells on glass produced fewer than normal podosome belts and a greater number of podosome rings. Inhibition of excess RhoA activity could rescue this phenotype. Microtubular networks were also disrupted, consistent with previous findings of inverse relationships between Rho activity and microtubule stability [91,92]. In contrast, siRNA-treated osteoclasts on bone had normal sealing zone morphologies, as measured by sealing zone number, size, and overall shape [27]. Nonetheless, bone resorption by these cells was markedly inhibited; although the cells could produce normal numbers of resorption pits, the pits were decreased in size relative to control cells. This was due to the excess RhoA activity in siRNA-treated cells, since treatment with a RhoA inhibitor could reverse this effect. This defect is likely to be explained by the siRNA-treated cells having altered distribution and diminished activation of Src [27], a kinase that is necessary for ruffled border formation and resorptive capacity in osteoclasts [93,94,95]. The precise link between Myo9b/RhoA and Src signaling in osteoclasts is unclear, but in other cell types RhoA has been shown to recruit Src to its proper distribution in focal adhesions [96]. In macrophages, the transcription factor KLF5 was shown to activate Myo9b transcription, leading to increased podosome formation and cell migration [97]. However, mature osteoclasts appear to express little to no KLF5 [98], so regulators of Myo9b expression in osteoclasts are still unknown.

Recent studies demonstrated that Myo9b knockout mice have significant bone defects, including diminished bone size, trabecular and cortical bone parameters, and strength and stiffness [99,100]. Further, osteoclasts cultured from the knockout mice showed similar defects in resorption as siRNA-treated cells from the earlier study. However, overall bone resorption in the knockout mice, as measured by serum CTX-1, was not different from that of wild-type mice. Instead, the bone phenotype appeared to be caused by defective bone growth resulting from diminished osteoblastic responses to IGF-1 [100]. It may be that the resorption defects seen in vitro were not of sufficient magnitude to affect overall resorption in vivo. Alternately, endogenous cytokines may have been able to correct osteoclast in vivo. It was demonstrated that tumor necrosis factor alpha (TNFα), which interacts with Rho signaling pathways and can promote bone resorption, could correct defects in cultured cells lacking Myo9b [27]. Nonetheless, these studies demonstrate that in osteoclasts, defects caused by loss of Myo9b are due to dysregulation of RhoA signaling, consistent with findings from other cell model systems [101,102,103]. Therefore, Myo9b’s predominant role appears to be as a motorized signaling molecule that can regulate actin dynamics in a localized, targeted manner. 

## 6. Perspectives and Future Studies

Understanding of myosin function in osteoclasts necessitates the identification of all myosins expressed in these cells. Most of the studies of myosins in osteoclasts so far have been initiated using a bottom-up hypothesis-driven approach. That is, current understanding of myosin function was coupled with knowledge of the osteoclast cytoskeleton to drive exploration of targeted myosins. However, given that the human genome contains 38 myosin genes, individual motor proteins are likely to play roles in osteoclast biology that are difficult to predict a priori. Because of this, a more broad-based top-down approach can be useful in determining what other myosins are critical to osteoclast formation and function, and ultimately, to bone health. For example, transcriptome databases may provide clues to what myosins play key roles in osteoclast function. The BioGPS Mouse Cell Type and Tissue Gene Expression database [104] lists several myosins (Myo1e, Myo1f, Myo7a, and Myo9b) as being highly expressed in osteoclasts relative to other tissues, suggesting that some of these (like Myo9b) are important to osteoclast physiology. Indeed, both Myo1e and Myo1f were identified as components of macrophage podosomes through proteomic analysis and are therefore almost certain to be present in osteoclast podosomes [105]. These are closely related molecules referred to as long-tailed myosin I due to the presence of two TH (tail homology) domains not present in the remaining class I myosin members. Clues to the potential function of Myo1e in osteoclasts come from studies of this myosin in invadosomes (a term that includes both podosomes and related structures found in cancer cells). In virally transformed baby hamster kidney cells, Myo1e was shown to be present in the invadosome actin core and its presence there was directed by one of the TH domains (TH2) present in its long tail. Further, Myo1e was recruited to the plasma membrane at the position of newly forming invadosomes through another TH domain (TH1) and may serve as a scaffolding protein between the plasma membrane and F-actin during invadosome/podosome assembly [106]. It is likely that Myo1e serves a similar function in osteoclasts. Mining of current databases and generation of new datasets are likely to provide additional clues to which myosins are strong candidates for future studies. Although the myosins investigated so far have played roles in osteoclast-specific processes such as precursor fusion and podosome patterning, myosins can affect processes throughout the cell due to their varied structures and activities. It is almost certain that osteoclasts utilize myosins for processes that are as yet unidentified or unexplored. As more of these are brought to light, myosins may be attractive targets for pharmaceutical intervention. Although the use of pharmaceuticals to correct overactive or underactive myosins is still relatively unexploited [107], the current existence of class-specific small molecule inhibitors and activators suggests that continuing development of these drugs could be beneficial for regulation of osteoclast activity and for improved therapeutic benefit.

In addition to determining the complement of myosins expressed by osteoclasts, understanding how these motor proteins are regulated will be necessary to fully comprehend their cellular roles. A completely unexplored aspect of osteoclast myosin biology is the regulation of these myosins by their light chains. As briefly described above, myosin light chains associate noncovalently with heavy chains at IQ domains and regulate both the structural integrity and functionality of the myosin [108]. These may be of particular importance in unconventional myosins where calmodulin is present as a light chain (e.g., Myo10, Myo9b), given that calcium/calmodulin signaling plays critical roles in osteoclast formation, activity, and apoptosis [109]. Calmodulin (CaM) acts as an intracellular calcium-binding calcium sensor that can bind hundreds of protein targets and thus affect a broad array of signaling pathways. Osteoclasts in particular are exposed to a wide range of extracellular calcium concentrations ([Ca^2+^]_o_) due to bone’s function as the major bodily reserve of calcium. During the process of resorption when bone mineral is released, the concentration of free calcium in the resorption pit may reach as high as 40 mM (compared to about 1 mM during normal homeostasis) while the remaining osteoclast surface encounters calcium levels of less than 2 mM [110,111]. High [Ca^2+^]_o_ serves as a negative feedback mechanism on osteoclasts, as it has been demonstrated to inhibit their formation, induce apoptosis, and cause modifications in the cytoskeleton that inhibit bone resorption [109]. These activities appear to require the calcium-sensing receptor (CaSR), a G-protein-coupled receptor that is also highly expressed in parathyroid and kidney [112]. Sensing of high [Ca^2+^]_o_ stimulates increased intracellular calcium, leading to diverse changes in cytoskeletal-mediated morphology and function such as cell retraction, loss of podosomes, and inhibition of bone resorption [113,114,115,116]. Calmodulin is likely to play a role in these effects, since attachment of osteoclasts to bone triggers increased CaM expression at the bone–osteoclast interface [117] where calcium levels can vary broadly. This suggests that cytoskeletal elements involved in bone resorption may be particularly sensitive to calcium-calmodulin signaling. Unfortunately, the functional roles of CaM in osteoclasts have not been widely studied, and nothing is known about how CaM may act as a light chain to affect myosin activity in these cells [118]. In addition to regulating activity of mature osteoclasts, it is also likely that calmodulin regulation of myosins may play roles in cell differentiation. Further, CaM signaling also may involve myosins in ways other than acting as light chains. This was recently shown in a study of calcium signaling during late osteoclast differentiation. The calcium channel TRPV4 (transient receptor potential vanilloid 4) was shown to be necessary for terminal differentiation and to regulate calcium-calmodulin signaling. Notably, Myo2a was shown to bind this channel and be necessary for its activation and subsequent differentiation of the cells [119].

The studies reviewed here have only begun to reveal the varied roles of myosins in osteoclast biology. While Myo2a has received most of the attention for its functions in podosome patterning and osteoclastogenesis, other myosins known or suspected to be expressed in osteoclasts (e.g., Myo2b, Myo2c, Myo1e, Myo1f, Myo7a) have so far gone unstudied in these cells. In addition, myosins of other classes are certain to be expressed in osteoclasts. Many of the unusual and unique properties of these cells, including their formation by fusion, migratory properties, and sealing zone formation, are results of a highly dynamic actin cytoskeleton. As the molecular motors associated with this dynamic actin, myosins can contribute in myriad ways to osteoclasts’ remarkable cellular functions. Exploration of their roles will undoubtedly illuminate more unusual aspects of osteoclast biology and may provide targets for therapeutic manipulation of osteoclast function.
